# Retraction: Pharmacological Inhibition of Microsomal Prostaglandin E Synthase-1 Suppresses Epidermal Growth Factor Receptor-Mediated Tumor Growth and Angiogenesis

**DOI:** 10.1371/journal.pone.0352619

**Published:** 2026-06-30

**Authors:** The PLOS One Editors

After this article [1] was published, concerns were raised regarding panels in Fig 4. Specifically,

- The mPGES1 panel of Fig 4D in [1] appears similar to the iNOS panel of Fig 6A in [2].
- Lanes 1-2 and 6-7 of the EGFR panel of Fig 4A in [1] appear similar to each other.
- Lanes 3 and 6 of the P-Tyr panel of Fig 4A appear similar to each other.

Corresponding author SD stated that they were unable to retrieve the original images underlying the P-Tyr panel of Fig 4A or the mPGES1 panel of Fig 4D. The underlying image provided for the EGFR panel of Fig 4A did not appear to match the published panel, and other underlying blots provided raised further concerns with the results in Fig 4.

In light of the above concerns that question the integrity and reliability of the reported results and conclusions, the *PLOS One* Editors retract this article [1].

FF and SD did not agree with the retraction. ET, EB, IC, LP, GM, MAA, NC, AG, and MZ either could not be reached or did not respond directly.
